# Lithium Treatment Increases FKBP5 Protein but Not mRNA Expression in the Pituitary Gland of Depressive-like Rats

**DOI:** 10.3390/brainsci15040389

**Published:** 2025-04-10

**Authors:** Mikołaj Kubiak, Wiktoria Majewska, Maria Kachel, Antonina Dola, Weronika Koga, Joanna Nowakowska, Wojciech Langwiński, Aleksandra Szczepankiewicz

**Affiliations:** 1 Molecular and Cell Biology Unit, Poznan University of Medical Sciences, 60-806 Poznan, Poland; mikolaj.kubiak@igcz.poznan.pl (M.K.); majewska.wiktoria.00@gmail.com (W.M.); maria00kachel@gmail.com (M.K.); toskadola@gmail.com (A.D.); weronikakoga@gmail.com (W.K.); asianowakowska781@gmail.com (J.N.); wlangwinski654@gmail.com (W.L.); 2 Department of Medical and Clinical Genetics, Institute of Human Genetics, Polish Academy of Sciences, 60-179 Poznan, Poland; 3 Centre of Experimental Medicine, Poznan University of Medical Sciences, 60-806 Poznan, Poland

**Keywords:** chronic mild stress, depression model, FKBP5 expression, lithium treatment, microRNA, pituitary

## Abstract

**Background**: Depression is a common psychiatric disorder that may be caused by dysregulation of the hypothalamic–pituitary–adrenal (HPA) axis. The glucocorticoid receptor (GR) plays a significant role in regulating this axis. One negative regulator of GR action, previously associated with depressive behavior, is the overexpression of FK506-binding protein 5 (FKBP5), which may be regulated by microRNAs, including miR-511-5p. **Objectives**: In a rat model of depression, we aimed to investigate the expression of Fkbp5 and its regulator, miRNA-511-5p, during short- and long-term lithium treatment in four brain regions: the hypothalamus, hippocampus, pituitary, and frontal cortex. **Methods**: We used a rat model of depression induced by chronic mild stress (CMS) to assess if short- and long-term lithium treatment (7 and 42 days) influences *Fkbp5* expression in the brain. We also assessed the effects of lithium treatment on the blood levels of corticosterone in CMS-exposed rats as compared to control groups. The changes in the expression of Fkbp5 were assessed by qPCR and Western blot. The expression of rno-miR-511-5p was assessed using qPCR. Statistical analysis was conducted in GraphPad Prism 9. **Results**: We found that long-term lithium treatment increased the expression of the FKBP5 protein in the pituitary gland in the lithium-treated rats compared to the control group. We also observed significant changes in Fkbp5 mRNA levels between CMS-exposed rats compared to the control animals, without significant changes in mRNA levels observed during short- and long-term lithium treatment in any brain region. We found no expression of rno-miR-511-5p in the analyzed brain regions. The corticosterone levels were significantly higher in CMS-exposed rats compared to the control, with no significant changes found between lithium-treated and control rats. **Conclusions**: FKBP5 protein expression in the brain may be regulated by long-term lithium treatment, thus affecting GR signaling in the rat depression model.

## 1. Introduction

Depression is one of the most prevalent psychiatric disorders. The COVID-19 pandemic increased the prevalence of depressive and anxiety disorders by 25%. Multiple factors during the pandemic period, such as health anxiety, economic recession, and loneliness caused by social isolation, resulted in chronic stress, and the increase highlighted the need for better and more efficient methods of diagnosis and treatment of depression [1,2].

The pathophysiology of depression is complex and involves aberrant neurotransmitter release, the response of the immune system, and hypothalamic–pituitary–adrenal (HPA) axis dysregulation in response to chronic stress. A recent genome-wide association (GWA) study showed the importance of the interplay between environmental risk factors such as chronic stress and the predisposing genetic variants in the pathophysiology of depressive disorders. Single-nucleotide polymorphisms and pathogenic gene variants within the HPA axis associated with depression together with exposure to chronic stress increase the risk of depression [3]. Chronic stress in childhood, during intense brain development, previously correlated with smaller volumes of the hippocampus and frontal cortex and larger volumes of the amygdala, thus changing neural connections and impeding the brain’s adaptiveness to stressful situations [4].

**Figure 1:**
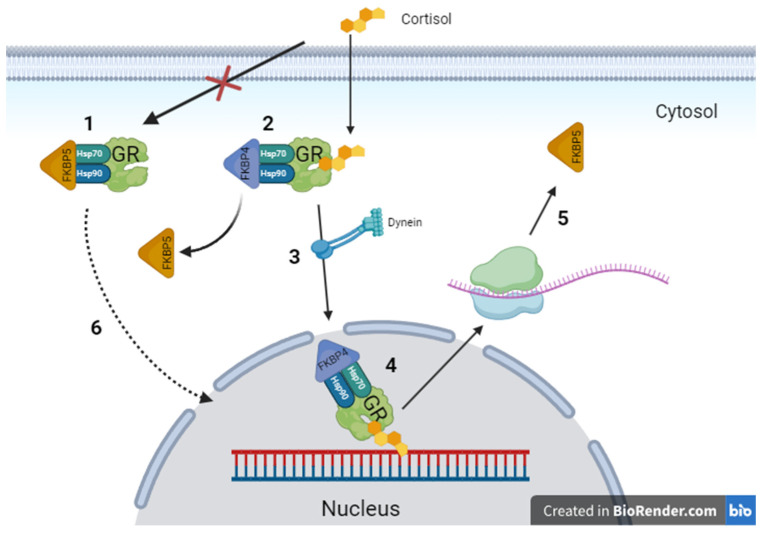
Mechanism of FKBP5 in the regulation of the glucocorticoid receptor. 1—FKBP5 stabilizes the GR complex, allowing other chaperones to join the complex, allowing for its maturation. 2—FKBP5 decreases the GR affinity to cortisol. By exchanging FKBP5 for FKBP4, cortisol binds with GR. 3—GR bound with cortisol is transferred by dyneins to the nucleus. 4—A fully formed complex binds with DNA, allowing for the transcription of genes associated with stress response. 5—Active GR increases the levels of FKBP5 expression, resulting in a negative feedback loop. 6—FKBP5 plays a role in the nucleus transfer of inactive GR complexes, resulting in their downregulation. FKBP5—FK506-binding protein 5, FKBP4—FK506-binding protein 4, Hsp90—heat shock protein 90, Hsp70—heat shock protein 70, GR—glucocorticoid receptor.

Chronically elevated stress hormone levels (cortisol, corticosterone) can dysregulate the HPA axis and response to stress. The HPA axis controls the release of cortisol or corticosterone, which interacts with the glucocorticoid receptor (GR) localized in the brain. Cortisol mostly acts in the hippocampus, whereas negative feedback actions are mediated at the level of the pituitary and other activated brain regions [5,6]. The impaired negative feedback loop of the GR is one of the underlying causes of mood disorders due to partial glucocorticoid resistance [7]. FK506 binding protein 5 (FKBP5) is a co-chaperone of GR that plays a significant role in forming the receptor complex. The active complex, bound to cortisol, is transferred into the nucleus, where GR stimulates the expression of genes associated with stress response. FKBP5 is also associated with the nuclear transfer of the inactive beta-isoform of GR, decreasing its number in the cytoplasm, resulting in its downregulation and an overall decrease in and inhibition of GR signaling [8,9,10]. FKBP5-based regulation of GR plays a significant role in the cellular response to cortisol, which is a part of the overall stress response (Figure 1).

Considering the role of FKBP5 in regulating the GR and stress response function, previous studies analyzed its expression in animal models of depression. Hausl et al. found that the overexpression of FKBP5 in the hypothalamus of mice led to enhanced stress response, confirmed by higher corticosterone blood levels in both groups of animals, exposed and not exposed to chronic stress [11]. Similarly, the overexpression of FKBP5 corresponded with lower neuron density in the hippocampus in mice exposed to early-life stress and increased susceptibility to depression-like behaviors [12]. FKBP5 also showed increased expression in the pituitary in a mouse model of psychosocial stress using chronic subordinate colony housing, whereas *Fkbp5* knockout mice in corticotrope cells of the anterior pituitary showed improved negative feedback and decreased hyperactivity of the HPA axis compared to wild-type mice, pointing at a possible inhibitory role of FKBP5 in stress regulation [11,13]. Häusl et al. found that the overexpression of *Fkbp5* in the paraventricular nucleus of the hypothalamus (PVN) of mice caused a significant overactivation of the HPA axis. The study also found that the overexpression of *Fkbp5* in specific neuron cells producing corticotrophin-releasing hormone (CRH) *Crh+* cells partially recapulated the effects of *Fkbp5* overexpression in PVN. This study showed that FKBP5 can act as an inhibitor of the HPA axis through interactions with specific hormone-producing neurons of the hypothalamus [11].

The overexpression of FKBP5 correlated with depression-like symptoms in rat models of depression, along with differential expression of microRNAs (miRNAs) involved in *Fkbp5* regulation, such as miR-511-5p. This miRNA binds with the 3′UTR region of the *Fkbp5* gene, thus suppressing its expression [14].

In depressive patients, a previous study found that antidepressant treatment decreased FKBP5 expression in peripheral blood cells, highlighting the link between the expression of the co-chaperone and depression [15]. The expression of FKBP5 influences the effectiveness of multiple antidepressant and mood-stabilizing drugs [16]. Previous studies found a correlation between the expression of genes involved in stress regulation, such as FKBP5 and CRHR1, and response to lithium treatment in bipolar disorder and major depressive disorder (MDD) [17]. Although lithium has been used as a mood stabilizer and also in drug-resistant depression, its exact molecular mechanism on HPA axis regulation and stress response is not fully understood [18,19].

This study aimed to determine the changes in the expression of *Fkbp5* and its validated regulator, miRNA-511-5p, during short- and long-term lithium treatment in four brain regions: the hypothalamus, hippocampus, pituitary, and frontal cortex in a chronic mild stress rat model of depression.

## 2. Materials and Methods

### 2.1. Rat Model of Depression

**Figure 2:**
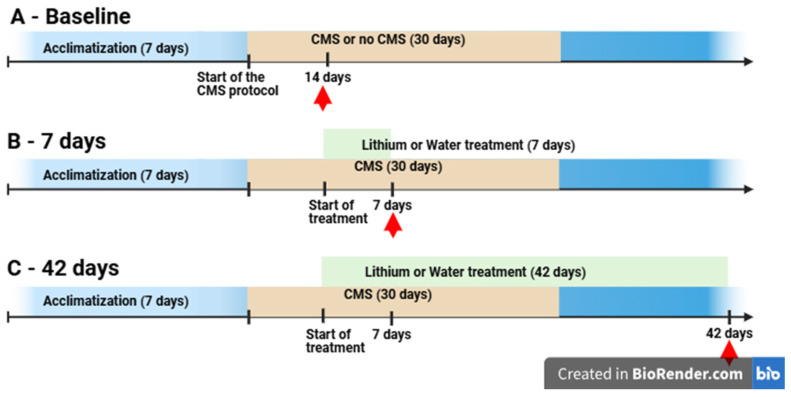
Outline of the animal experiment as performed by Szczepankiewicz et al. [22]. To induce depression in rats, the animals underwent the chronic mild stress (CMS) protocol, and after two weeks, the animals started lithium treatment or water administration. (**A**) Baseline model of depression—rats that underwent the CMS protocol for 14 days, non-stressed rats—control group; (**B**) 7 days—short-term lithium treatment: rats that underwent the CMS protocol and were treated with lithium or water for 7 days; (**C**) 42 days—long-term lithium treatment: rats that underwent the CMS protocol and were treated with lithium or water for 42 days. Red arrows mark the time points when the samples were collected.

Brain tissues were obtained from male Wistar rats weighing 180 ± 10 g, euthanized for the previous experiment. The animal study protocol was approved by the local ethical committee at Poznan University of Life Sciences, Poland (agreement no. 22/2017, 23 June 2017). The animals were housed 5 per cage at a temperature maintained at 22 ± 1 °C, with food and water ad libitum. A 12 h light/dark cycle was maintained, with lights turning on at 7:00 a.m. The experiments and material collection were performed at the same time (10–11 a.m.) for each group to avoid potential circadian variations. The CMS protocol was developed by Wilner et al. to simulate symptoms of depression found in humans [20]. The animal model was performed according to the Papp protocol [21]. The rats underwent two weeks of exposure to constant mild stressors (e.g., food and water deprivation, cage tilting, intermittent illumination, soiled cage) that resulted in behavioral changes. Animals were randomly divided into groups for each time point of the experiment: CMS rats—rats after four weeks of stress exposure (according to the chronic mild stress protocol) (*n* = 8) and non-stressed rats (rats not exposed to stress) (*n* = 6); 7 days—to assess short-term lithium effects, rats were exposed to CMS for two weeks and then received lithium or the vehicle (water) for 7 days (*n* = 3 in each group); 42 days—to assess chronic lithium effects, rats were exposed to CMS for two weeks and then received lithium or the vehicle (water) for 42 days (*n* = 3 in each group) (Figure 2, Table 1). Lithium was administered orally with a syringe as described previously [22].

### 2.2. Molecular Analysis

After decapitation, four brain regions (the frontal cortex, pituitary, hippocampus, and hypothalamus) were collected and snap-frozen in liquid nitrogen. Tissues were stored at −80 °C until further procedures. According to the manufacturer’s protocol, total RNA and miRNA were extracted using the ExtractMe miRNA kit (Blirt, Gdansk, Poland). RNA concentration was measured using Nanodrop. We examined RNA integrity (RIN) using Tape Station 2200 (Agilent, St. Clara, CA, USA). Samples with RIN > 8 were used in this study. Protein was isolated from the same brain tissues using RIPA buffer (Thermo Fisher, Waltham, MA, USA). Extracted materials were stored at −80 °C.

Total RNA from all samples underwent reverse transcription using a Go-Script reverse transcription kit (Promega, Waldorf, Germany), according to the manufacturer’s protocol. MiRNA levels were quantified using a Qubit microRNA Assay Kit (Thermo Fisher, USA), and reverse transcription was carried out using TaqMan Advanced miRNA Assay kit (Thermo Fisher, USA). Then, the expression of Fkbp5 mRNA was analyzed using GoTaq qPCR Master Mix (Promega, Poland) and specific Fkbp5 primers (forward: 5′ CAG AGC AGG ATG CCA AGG 3′ and reverse: 5′ GCG TCA TAC GTG GCC TTC 3′), with Gapdh as a reference gene (forward: 5′ CAC TCC CTC AAG ATT GTC AGC AA 3′ and reverse: 5′ GGC ATG GAC TGT GGT CAT GA 3′). Mir-511-5p expression was quantified using a TaqMan Advanced miRNA assay (Thermo Fisher, USA, assay ID: Hs03303847_pri) and mir-26a (assay ID: Hs03303430_pri) as an endogenous control. The qPCR was performed in a 7900HT Real-Time PCR instrument. The expression of the housekeeping gene was confirmed to be stable across all the studied samples.

### 2.3. Protein Analysis

Protein concentration was measured using a Pierce BCA Protein Assay Kit (Thermo Fisher Scientific). For the Western blot, we used 10 μg of total protein according to the routine procedure used in our laboratory [23]. Samples were mixed with Tris–Glycine SDS Sample Buffer (2×) (Thermo Fisher Scientific) and NuPAGE Sample Reducing Agent (10×) (Thermo Fisher Scientific), denatured in 85 °C for 2 min, and used for SDS-PAGE gel electrophoresis. We stained the gel with No-Stain Protein Labeling Reagent (Thermo Fisher Scientific) for total protein normalization. Proteins were transferred to a nitrocellulose membrane using an iBlot 2 Dry Blotting (Thermo Fisher Scientific) system. The membrane was then blocked in PBS and incubated overnight with rabbit monoclonal anti-FKBP51 (ab126715) primary antibody (1:1000) at 4 °C. The membrane was washed three times for 10 min with PBST, followed by incubation with anti-rabbit IgG HRP-conjugated (HAF008) secondary antibody (1:1000) for 1 h at room temperature. The membrane was washed three times for 10 min with PBST, followed by protein band detection with ECL SuperSignal West Pico PLUS Chemiluminescent Substrate (Thermo Fisher Scientific) in ChemiDoc Imaging System (Bio-Rad, Hercules, CA, USA). The band and total protein intensities were measured in ImageJ version 1.54.

### 2.4. Corticosterone Analysis

To assess the concentration of corticosterone in the rat serum for each time point, we used an ELISA kit (LDN). Blood samples for serum were taken from tubes without anticoagulants between 9 and 10 a.m. Blood was then centrifuged to obtain serum, and samples were frozen at −80 °C for further analysis. Corticosterone was measured in undiluted samples according to the manufacturer’s protocol. The absorbance was read on a plate reader at a 450 nm wavelength (Asys UVM 340). Corticosterone concentration was quantified against a standard curve calibrated with known amounts of protein (standard).

### 2.5. Statistical Analysis

Statistical analysis was performed in GraphPad Prism. Data were analyzed using the *t*-test after the confirmation of normal data distribution using the Shapiro–Wilk test. We compared the expression levels at baseline between rats exposed to stress versus control rats and between the lithium-treated groups (after 7 and 42 days) and the corresponding control group (without lithium). A one-way ANOVA test was used to analyze the changes in the expression of mRNA and protein levels of FKBP5 throughout the experiment. A cut-off value of α = 0.05 was used to determine the statistical significance of the results. Data on graphs are shown as mean ± standard deviation (SD).

## 3. Results

### 3.1. Fkbp5 Expression Is Significantly Increased in the Hypothalamus and Frontal Cortex of Stressed Rats

**Figure 3:**
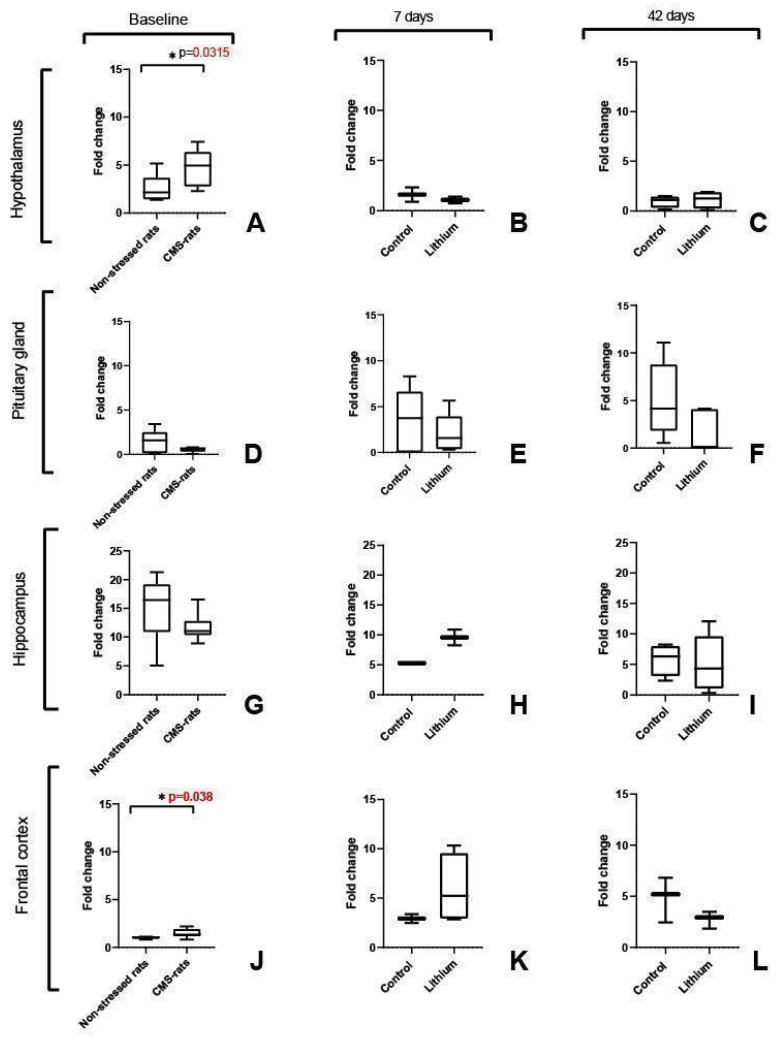
The relative expression of Fkbp5 in stressed rats (**A**–**L**). Expression levels are presented by fold change (2^−ΔΔCt^) regarding the reference gene (Gapdh). Baseline—CMS rats (underwent the chronic mild stress protocol), *n* = 8; non-stressed rats (control), *n* = 6; 7d—7 days of treatment, *n* = 6; 42d—42 days of treatment, *n* = 6; control for lithium groups—rats administered with water.

We found significantly higher Fkbp5 expression in the hypothalamus (*p* = 0.0315) and frontal cortex (*p* = 0.038) of rats exposed to chronic stress, as compared with the control group (Figure 3A,J). We did not find significant differences at the baseline for the other analyzed brain regions (pituitary and hippocampus). When we analyzed Fkbp5 expression after short- and long-term lithium administration, we did not observe significant differences in any brain region between the lithium-treated group and the control group (Figure 3B,C,E,F,H,I,K,L).

### 3.2. Mir-511-5p Expression Was Not Detected in the Rat Brain Regions

The expression of rno-miR-511-5p in the studied rat brain regions was not detected by qPCR in any experimental group of the studied regions: the hypothalamus, hippocampus, pituitary gland, and frontal cortex (no amplification until 40 cycles), whereas the endogenous control (rno-miR-26a) was detected in all samples and regions with a mean Ct value of 24.

### 3.3. FKBP5 Protein Significantly Increased During Long-Term Lithium Treatment in the Pituitary

**Figure 4:**
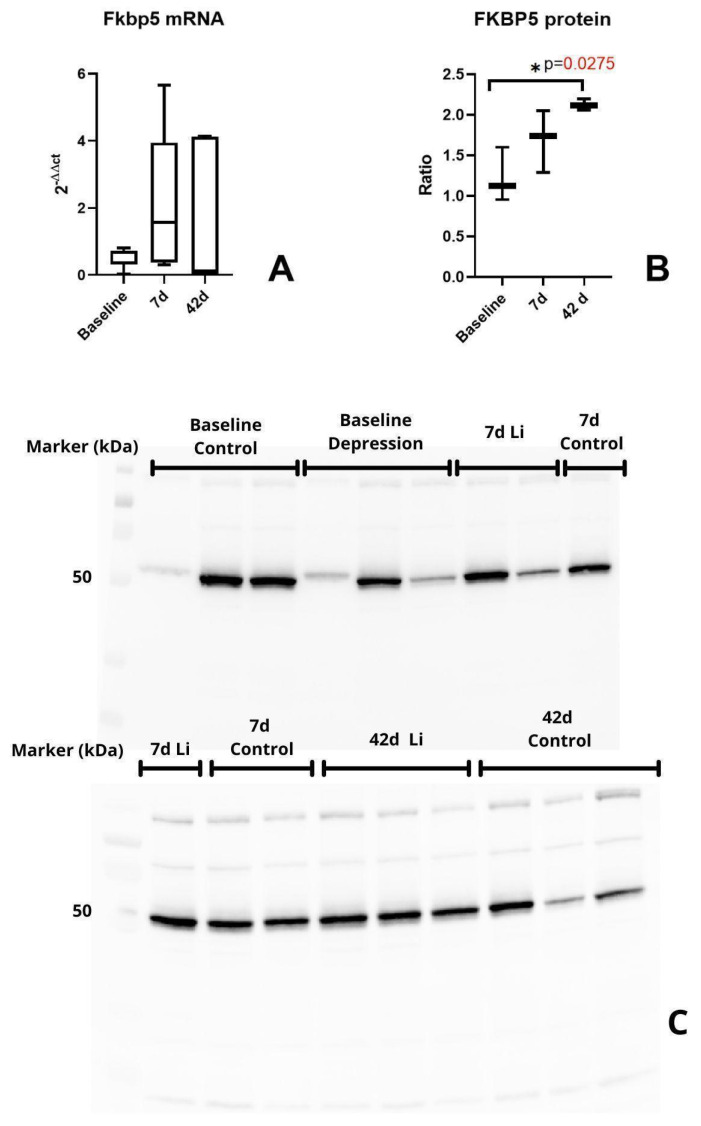
Fkbp5 expression in the pituitary. All samples were analyzed in triplicates. Levels of Fkbp5 mRNA (**A**). Results of Western blot analysis; a comparison of FKBP5 protein levels in the pituitary gland at different time points (**B**). Samples used in total protein normalization (**C**). Protein levels were presented as a ratio regarding total protein normalization. Baseline (changes in expression between CMS and control rats), *n* = 3; 7d—changes in expression between CMS rats treated and not treated with lithium at 7 days, *n* = 3; 42d—changes in expression between CMS rats treated and not treated with lithium at 42 days, *n* = 3.

Based on the primary role of the pituitary in the HPA axis regulation and its feedback loop, we decided to analyze the protein levels of the FKBP5 in this brain region. Western blot analysis of the protein levels of FKBP5 showed a significantly increased expression after chronic lithium treatment (*p* = 0.027) (Figure 4B).

### 3.4. Chronic Mild Stress and Long-Term Lithium Treatment Affect Corticosterone Levels

**Figure 5:**
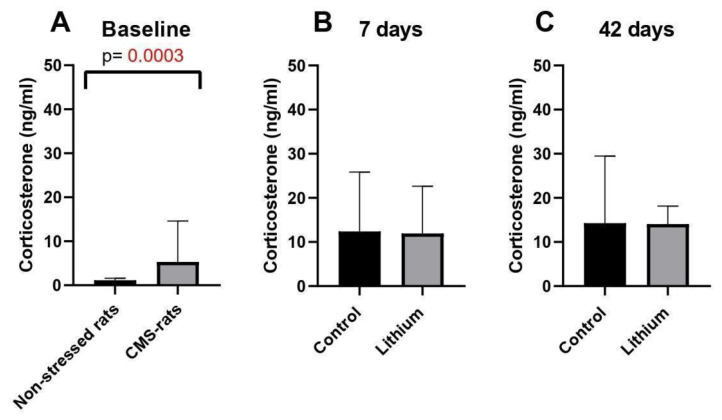
Corticosterone level comparison between the groups (**A**–**C**). Baseline—CMS rats (underwent the chronic mild stress protocol), *n* = 8, non-stressed rats (control), *n* = 6; 7d—7 days of lithium treatment *n* = 6; 42d—42 days of lithium treatment, *n* = 6, control for lithium groups—rats administered with water.

As we described previously, we found significant differences in the behavior of rats exposed to stress compared to the control. We did not observe significant changes in behavior after lithium administration [24]. Therefore, in this study, we analyzed if stress exposure and lithium influence corticosterone levels in the serum of rats at different time points (baseline, 7 days, 42 days). We observed significantly increased corticosterone levels in rats exposed to stress compared to the control group (Figure 5A). During short- and long-term lithium administration, we did not observe significant differences between groups (Figure 5B,C); however, the corticosterone levels showed a decreasing trend in the rats receiving lithium for 42 days in comparison to those receiving the vehicle (*p* = 0.06) (Figure 5C)

## 4. Discussion

Our study showed that long-term lithium treatment increased the expression of the FKBP5 protein in the pituitary compared to the control group. We also observed significant changes in *Fkbp5* mRNA levels at baseline in the frontal cortex and hypothalamus between CMS-exposed rats compared to the control animals. Still, no significant changes were observed during short- and long-term lithium treatment in the analyzed brain regions.

In the pituitary gland, we observed a significant increase in FKBP5 protein levels after 42 days of lithium treatment compared to the control group, indicating that FKBP5 protein expression in the pituitary gland may be affected by long-term lithium treatment in an animal model of depression. A previous study by Ising et al. showed that the upregulated expression of FKBP5 occurred in patients unresponsive to antidepressant treatment, while downregulation was observed in patients responding to treatment [15]. A study analyzing the effects of both the CMS protocol and antidepressant treatment on the expression of *Fkbp5* and GR in a rat model of depression showed similar differences between mRNA and protein levels. Guidotti et al. found that mRNA and protein levels of *Fkbp5* only partially mirror each other in the hippocampus, prefrontal cortex, and hypothalamus of CMS rats, both before and after treatment. The researchers used duloxetine, a serotonin–norepinephrine reuptake inhibitor (SNRI) commonly used in treating MDD in patients. Of note is that the treated rats showed a decrease in *Fkbp5* mRNA levels in the ventral hippocampus while exhibiting an increase in FKBP5 protein levels. This mirrors our study’s results observed in the pituitary gland [25]. Daily changes in cortisol levels can significantly and almost immediately affect the expression levels of FKBP5 in the blood of healthy humans. This change was observed 90 min after blood cortisol levels increased. This relationship may impact mRNA and protein levels of FKBP5 differently due to the temporal relationship between transcription and translation. This can explain the discrepancy between *Fkbp5* mRNA and protein levels in the pituitary gland of lithium-treated depressive-like rats, as observed in our study [26]. Moreover, taking into account that the major sites for this GC-mediated negative feedback inhibition are in the anterior pituitary gland, which mediates the negative feedback loop in response to excessive steroid hormone levels, we suggest that increased FKBP5 expression in the pituitary upon long-term lithium treatment may exert an inhibiting effect on GR signaling and desensitizing glucocorticoid action [27].

The findings regarding *Fkbp5* mRNA expression are in line with the previous studies. Zimmer et al. suggested that *Fkbp5* expression is related to HPA axis flexibility, which is responsible for appropriate response to stressors. They showed that lower FKBP5 mRNA expression in the hypothalamus was associated with higher HPA flexibility [28]. Another study of male mice with *Fkbp5* knockout in the paraventricular nucleus showed reduced plasma corticosterone levels after stress exposure compared to the control group (not exposed to stress). Additionally, they observed that mice overexpressing *Fkbp5* in the hypothalamus demonstrated a depressed phenotype, which further confirms the role of *Fkbp5* in depression [11]. As was stated previously, high levels of *Fkbp5* in the hypothalamus, and especially the PVN, are associated with depressive-like states and indicate an inhibitory role of this gene on the HPA axis (through the function of *Crh+* neurons) [11]. Our study confirmed increased *Fkbp5* expression at baseline in depressive rats, but no significant changes were observed after short-term (7 days) or long-term (42 days) lithium administration in this brain region.

In the hippocampus, we did not show any significant differences in the expression of *Fkbp5* at baseline between the rats exposed to stress and the control group. A study in transgenic mice overexpressing *Fkbp5* in the brain and exposed to early-life stress showed increased neuronal loss in the hippocampus by altering glial cells in the brain [12]. In our study, lithium treatment did not significantly affect *Fkbp5* expression, independent of treatment duration, in the hippocampus. A previous study analyzing the lithium effect in this brain region found that lithium augmented the impact of antidepressant desipramine and increased the ΔFosB/FosB-positive cell density in the hippocampus. ΔFosB is a marker of neuronal activity in response to stress and is also known to accumulate in brain regions after antidepressant therapy augmented with lithium [29]. However, the exact molecular mechanism by which lithium affects the hippocampus remains unknown.

Similarly to the hypothalamus, we observed higher *Fkbp5* expression in the frontal cortex in rats exposed to stress in comparison to the control group, thus suggesting that this brain region is also affected by stress response. Our observation is consistent with the previous study that found FKBP5 overexpression in this brain region on mRNA and protein levels in the autopsy samples of depressive HIV-infected patients [30].

Surprisingly, we did not detect the expression of mir-511-5p in any of the analyzed brain regions in our rat model of depression. The previous observations in mice indicated higher expression of miR-511-5p in the depression model. This discrepancy may result from differences between rats and mice regarding the expression of this miRNA [17]. However, our data from miRNA sequencing confirmed the lack of expression of miR-511-5p in the pituitary in the samples from the same rat depression model [22].

A previous study in humans found that lithium treatment decreased cortisol levels in patients suffering from major depressive or bipolar disorder, pointing to a possible link between lithium and the regulation of the HPA axis via this hormone [31]. Analysis of corticosterone levels in the blood showed significant differences between stress-exposed rats and the control group at baseline, thus confirming depressive-like changes in this stress hormone in rats exposed to chronic mild stress. After short- and long-term lithium treatment, we observed a slight decrease in corticosterone levels, but these changes were not significant. This observation may suggest a limited effect of lithium on HPA axis function in the rat model of depression. The discrepancy between our study and Smigan et al. [31] could be due to the intrinsic differences between humans and rats in terms of HPA axis regulation.

The corticosterone levels correlate well with behavioral changes observed in CMS rats treated with lithium. Significant differences were found in our previous study regarding behavior between non-stressed rats and CMS-exposed rats using the open-field test, thus confirming the effectiveness of the CMS protocol to generate depressive-like behavior in our rat model of depression [22]. After lithium treatment, the changes were not significant between treated and non-treated rats, although they showed an increasing trend in distance travelled upon lithium treatment. Similarly to corticosterone, CMS caused significant changes in the animal behavior, while lithium treatment did not significantly alter the studied parameters [22].

The limitation of our study may be the sample size, which could not have been sufficient to detect changes in corticosterone levels and *Fkbp5* gene expression between experimental groups. Additionally, the genetic and structural differences between humans and rats could also influence the effects of both lithium treatment and the overall reaction to stress. Thus, further studies are necessary to verify our findings.

## 5. Conclusions

Lithium treatment did not significantly affect the Fkbp5 expression in the rat brain, except in the pituitary, where chronic lithium treatment increased protein levels. Those observations correlated well with blood corticosterone levels at baseline, with limited response to lithium treatment. Our study highlights the need for further research on the role of lithium in stress response and Fkbp5 regulation.

## Figures and Tables

**Table 1:** Groups of animals used in this study.

Group	Number of Animals
Baseline—CMS (stress-exposed rats)	8
Baseline—control (non-stress-exposed rats)	6
7 days—lithium (CMS + lithium)	3
7 days—control (CMS + water)	3
42 days—lithium (CMS + lithium)	3
42 days—control (CMS + water)	3

## Data Availability

The original contributions presented in this study are included in the article. Further inquiries can be directed to the corresponding author.

## Abbreviations

The following abbreviations are used in this manuscript:

FKBP5	FK506-binding protein 5
HPA axis	Hypothalamic–pituitary–adrenal axis
GR	Glucocorticoid receptor
GWA	Genome-wide association
CMS	Chronic mild stress
PVN	Paraventricular nucleus
CRH	Corticotropin-releasing hormone
